# Use of a mobile workforce to improve recruitment across multiple sites within a local clinical research network

**DOI:** 10.1186/1745-6215-16-S2-P99

**Published:** 2015-11-16

**Authors:** Robert Hughes, Dawn Beaumont-Jewell, Rachel Taylor, Kylie Gyertson

**Affiliations:** 1 University College London Hospital, London, UK; 2 National Institute for Health Research Clinical Research Network, London, UK

## 

Patient recruitment to clinical research is an important metric used to assess the effectiveness of research departments and decide resource allocation. The Mobile Workforce (MW) has been put in place to increase recruitment to haematology studies across the North Thames region.

## Method

The NIHR North Thames division three has provided funding to employ a group of clinical trial practitioners and data managers. The team are employed by and based at large teaching hospitals and travel to other sites within the North Thames region to open and manage research studies with a high anticipated recruitment.

## Results

The MW has recruited over 200 patients to research projects in its first year of operation. This has contributed to a rise in the North Thames LCRN's haematology recruitment from 512 in 2013-2014 to 851 in 2014-2015. The MW has encouraged senior clinicians to participate in research and has facilitated the set-up of new research projects. The MW has reduced the need for staff training and has allowed for greater flexibility in meeting staffing requirements.

## Conclusion

The MW has achieved a significant increase in study accrual during its first year. The MW has allowed previously unsupported sites to participate in research and has caused an increase in investigator interest in research. The NIHR seeks to replicate the approach across divisions and regions to provide an efficient and cost effective approach to study recruitment.

